# Internet-based cognitive behavioural therapy programme as an intervention for people diagnosed with adult-onset, focal, isolated, idiopathic cervical dystonia: a feasibility study protocol

**DOI:** 10.1186/s40814-020-00641-x

**Published:** 2020-07-15

**Authors:** Megan E. Wadon, Mia Winter, Kathryn J. Peall

**Affiliations:** 1 Neuroscience and Mental Health Research Institute, Hadyn Ellis Building, Cardiff University, Cardiff, UK; 2 Department of Clinical Neuropsychology, University Hospital of Wales, Cardiff, UK

## Abstract

**Background:**

Dystonia is one of the most common forms of movement disorder, caused by the co-contraction of antagonistic muscles, leading to abnormal postures and considerable disability. Non-motor symptoms, notably psychiatric disorders, are well recognised comorbid features of the disorder. However, there is no standardised model for the management of these symptoms in dystonia, with them frequently going undiagnosed and untreated. An internet-based cognitive behavioural therapy programme may provide a future model of care that also maximises available resources.

**Methods:**

This study represents a two-armed randomised feasibility trail, aiming to recruit a total of 20 participants with a diagnosis of adult-onset primary focal cervical dystonia. Participants will be recruited from the Global Myoclonus Dystonia Registry and Dystonia Non-Motor Symptom Study (conducted at Cardiff University) based on presence of moderate symptoms of anxiety/depression as indicated by standardised questionnaires. All participants will undergo assessment at baseline, 3 and 6 months, with this including questionnaires for assessment of non-motor symptoms and clinical assessment of motor symptom severity. Participants will be randomised to either the control (*n* = 10) or treatment (*n* = 10) groups. The treatment group will be asked to complete one session of the online CBT program a week, for 8 weeks. The primary outcome measure will be the engagement of participants with the programme, with secondary outcomes of non-motor and motor symptom scores.

**Discussion:**

Promising results have been shown using face-to-face cognitive behavioural therapy to reduce levels of anxiety and depression in individuals with a diagnosis of dystonia. However, no studies to date have sought to determine the feasibility of an internet-delivered cognitive behavioural therapy programme. A number of effective internet-based programmes have been developed that combat anxiety and depression in the general population, suggesting the potential for its effectiveness in cervical dystonia patients. Success with this study would significantly impact the clinical care delivery for patients with cervical dystonia, as well as widening potential access to effective treatment.

**Trial registration:**

This feasibility trial has been registered with Health and Care Research Wales Research Directory. Trial registration number 44245. Date of registration: 21 November 2019. https://www.healthandcareresearch.gov.wales/research-studies-in-wales/

## Background

### Background and rationale

Dystonia is the third most common form of movement disorder, with an estimated prevalence of 1/900 (dystonia.org.uk). It involves co-contraction of antagonistic muscle groups leading to abnormal postures and movements, causing considerable disability. As a result, the disorder is associated with significant social, educational and health economic implications. There is also increasing recognition that non-motor symptoms form an important part of the disorder [1, 2]. A consistent psychiatric phenotype has been demonstrated across a number of cohorts, with up to 65.9% of individuals affected with symptoms including major depressive disorder and anxiety-related disorders [3–5]. Psychiatric symptoms, in particular anxiety and depression, have been shown to have a significant impact on the quality of life of individuals with cervical dystonia, and in some cases a larger impact on their physical quality of life than the severity of the dystonia itself [6–8].

Comorbid psychiatric symptoms in dystonia are frequently undiagnosed and largely untreated. There is no standardised model for the management of these symptoms, and for those who do receive treatment, this is often pharmacological, with many of the currently available treatments adversely affecting the underlying movement disorder [9]. Non-pharmacological interventions, such as psychological therapies, represent a potentially beneficial form of treatment, allowing primary management of the psychiatric symptoms while also benefitting overall motor function. Cognitive behavioural therapy (CBT) is a well-recognised psychotherapeutic approach that uses goal-orientated, systematic procedures to address cognitive processes and maladaptive behaviours, beliefs and emotions.

CBT has been shown to be particularly effective in the management of the psychiatric symptoms frequently observed in cervical dystonia [10, 11], with a recent feasibility study demonstrating the ability for face-to-face CBT combined with mindfulness to substantially reduce depression and anxiety scores using standardised questionnaires [12]. Unfortunately, timely access to face-to-face psychological therapy is often limited due to cost, waiting times, a shortage of suitably qualified therapists and the stigma associated with psychological therapy. As a result, a number of online, guided self-help CBT programmes have been developed, validated and shown to be successful in the management of anxiety and depression [13–15].

This study aims to demonstrate the feasibility of a validated online guided, self-help CBT programme in the management of non-motor and motor symptoms for patients with cervical dystonia. We believe this has the potential to provide a platform for a fully integrated, and cost-effective, model of care that could be implemented alongside currently available medical management, widening access to effective treatment and maximising the use of healthcare resources.

### Aims and objectives

This study is a two-arm randomised control feasibility study with the specific aim to determine the feasibility of a guided, internet-based self-help CBT programme in the management of psychiatric symptoms in patients with cervical dystonia. A secondary objective of this study is to explore the difference in non-motor and motor symptoms in patients with cervical dystonia that have completed an online CBT programme in comparison to those receiving routine clinical treatment of their motor symptoms alone.

#### Primary objective

(i) To assess the feasibility of using an online, self-guided, cognitive behavioural therapy programme for individuals diagnosed with cervical dystonia, considering the engagement of participants with the intervention, including the following:
  Response rates and adherence to the online CBT programme, including number of logins and modules completed
  The participants’ use of the optional resources included with the online CBT programme
  The willingness of eligible participants to receive the intervention and participate in a randomised control study, including the completion of follow-up appointments

#### Secondary objectives

(i) To explore if an online, self-guided CBT programme can reduce depression and anxiety levels in individuals with cervical dystonia.
(ii) To explore if improvement to anxiety and depression levels subsequent to the CBT program also have an impact on the motor symptoms observed in those with cervical dystonia.

### Study design

**Fig. 1:**
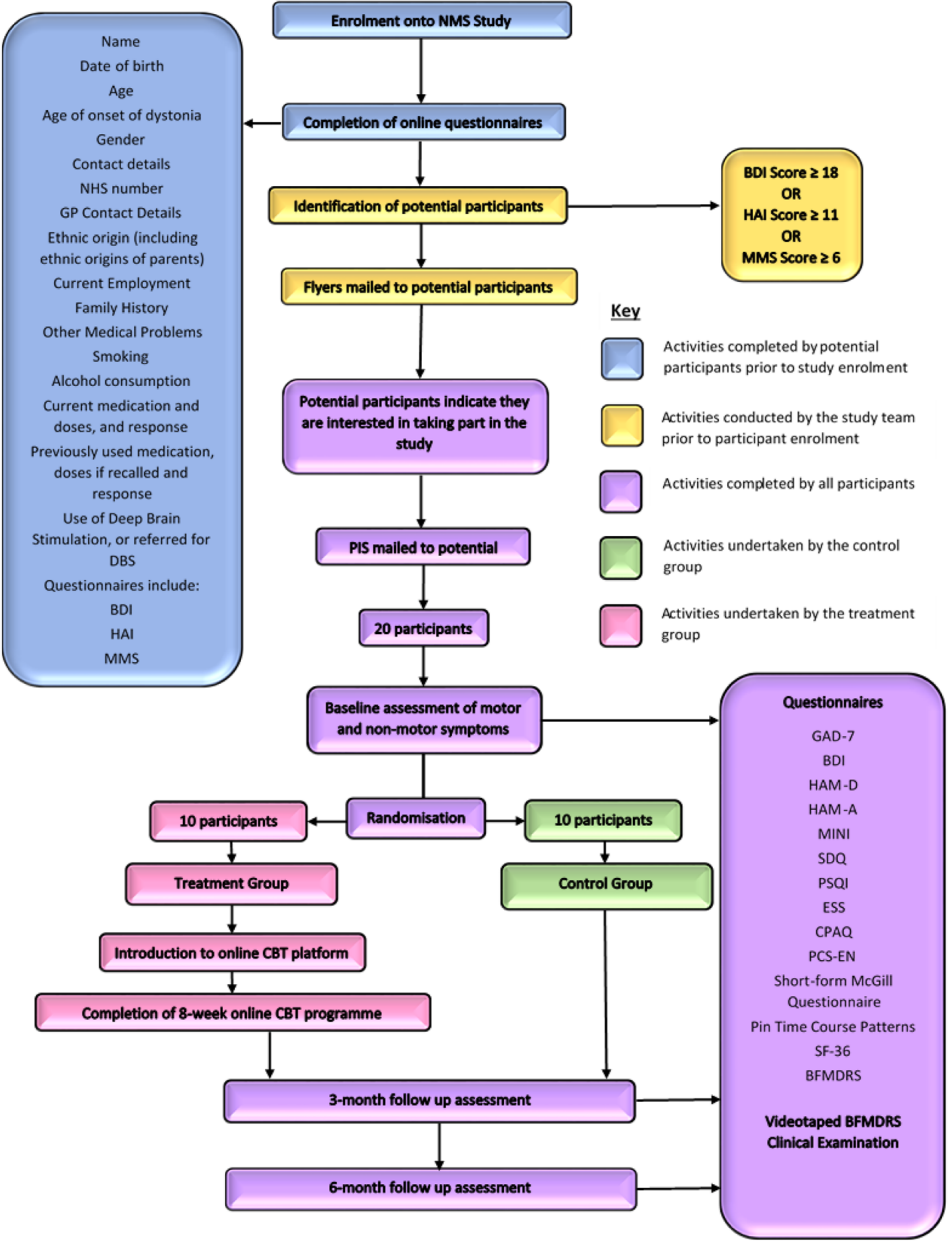
Schematic representation of feasibility study. BDI Beck’s Depression Inventory, BFMDRS Burke-Fahn-Marsden Dystonia Rating Scale, CBT cognitive behavioural therapy, CPAQ Chronic Pain Acceptance Questionnaire, DBS deep brain stimulation, ESS Epworth Sleepiness Scale, GAD-7 Generalised Anxiety Disorder-7, GP general practitioner, HAM-A Hamilton Anxiety Rating Scale, HAM-D Hamilton Depression Rating Scale, HAI Health Anxiety Inventory, MINI Mini International Neuropsychiatric Interview, MMS Modified Mini Screen, NHS National Health Service, NMS non-motor symptoms, PCS-EN Pain Catastrophising Questionnaire, PIS Participant Information Sheet, PSQI Pittsburgh Sleep Quality Index, SDQ Sleep Disorders Questionnaire, SF-36 Short Form-36 Health Survey

This study is a two-arm randomised feasibility study of a guided, internet-based self-help CBT programme for people with cervical dystonia, which also have elevated levels of depression and/or anxiety determined through the use of three clinically validated standardised questionnaires (Beck’s Depression Inventory, Health Anxiety Inventory, and Modified Mini Screen). All participants will undergo baseline assessments and assessments at 3 and 6 months that will take place either in the participants’ home or at our research clinic, dependent on the preference of the participant. These assessments will include a number of questionnaires relating to non-motor symptoms, including psychiatric symptoms, sleep disturbance and pain, as well as overall quality of life. A videotaped, standardised clinical examination will also be undertaken for assessment of their motor symptoms. This will allow for blinded rating of motor symptoms by two movement disorder experts. Those allocated to the control group will continue to receive only their routine clinical care for treatment of their motor symptoms, namely 3-monthly neurotoxin injections. Those allocated to the intervention will receive support setting up their account for the online CBT programme and receive guidance on how the programme works. The study schema is illustrated in Fig. 1.

## Methods

### Participants, interventions and outcomes

#### Study setting

Participants will be recruited from the Global Myoclonus Dystonia Registry and Dystonia Non-Motor Symptom Study (REC 18/WM/0031 IRAS project ID: 236219). Only individuals that have consented to be contacted about future studies will be contacted. Participants meeting the inclusion criteria for this study will be sent a study flyer inviting them to enquire further if they are interested in taking part in the research. A participant information sheet (PIS) will then be sent, and the potential participant will be invited to make an appointment at their convenience for their baseline assessment. Baseline assessment will include evaluation of the participant’s motor and non-motor symptoms as well as a standardised clinical examination (Table 1; Fig. 1). Baseline demographic information will already have been collected as part of the dystonia non-motor study (Fig. 1). This appointment will also include randomisation to either the treatment or the control arm.

**Table 1:** Study outcome assessments

Assessment	Description	Approximate duration	Type of data collected
*Psychiatric*			
Beck’s Depression Inventory	A 21-item questionnaire to identify mild mood disturbances, borderline clinical depression, moderate depression, severe depression or extreme depression. Freely available	2 min	Quantifiable
Hamilton Depression Rating Scale	A widely used and well-validated tool for measuring a patient’s depression, identifying mild, moderate, severe and very severe levels of depression	15–20 min	Quantifiable
Generalised Anxiety Disorder-7	A 7-item questionnaire to identify mild, moderate, or severe levels of anxiety. Freely available	< 1 min	Quantifiable
Hamilton Anxiety Rating Scale	A widely used and well-validated tool for measuring anxiety able to indicate mild, moderate, and severe levels of anxiety	15–20 min	Quantifiable
Mini International Neuropsychiatric Interview	An interview that covers a wide range of neuropsychiatric disorders including depression, panic disorders, anxiety, obsessive-compulsive disorder (OCD) and post-traumatic stress disorder (PTSD)	15–20 min	Yes/no
*Sleep*			
Sleep Disorders Questionnaire	A 16-item questionnaire that assesses insomnia, psychiatric disorders, circadian rhythm disorder, movement disorders and parasomnias. Freely available	1.5 min	Likert scale
Pittsburgh Sleep Quality Index	A questionnaire that measures sleep quality over a 1-month period, using 7 components to create a global score. Freely available	2–5 min	Likert scale
Epworth Sleepiness Scale	An 8-item questionnaire that measures the likelihood of dozing in different situations. Freely available	< 1 min	Likert scale
*Pain*			
Chronic Pain Acceptance Questionnaire	A 20-item questionnaire measuring the ability of the participant to accept their pain and carry on with daily activities despite the pain. Freely available	2 min	Quantitative
Pain Catastrophising Scale	A 13-item questionnaire that measures the participants’ thoughts and feelings about pain	2 min	Quantitative
Short-form McGill Questionnaire (pain description)	A questionnaire assessing the natures of the pain experienced by the participant. Freely available but copyright protected, requiring permission from Ronald Melzack for distribution	2 min	Likert scale
Pain Time Course Patterns	Participants select from a series of diagrams to indicate the best pattern of their pain experience during the course of the day	< 1 min	Qualitative
*Quality of life*			
Short-Form 36 Health Survey	A 36-item questionnaire that gathers information about the participants views about their health. Freely available	2 min	Quantitative
*Motor symptoms*			
BFMDRS Disability Scale	A patient completed report of disability in daily life activities. This is combined with an objective clinician scored standardised clinical examination to give the ‘gold standard’ tool for the assessment of dystonia severity	2 min	Likert scale (quantitative when combined with clinical examination score)
BFMDRS Clinical Examination	A standardised videotaped clinical examination of following the Burke-Fahn-Marsden Dystonia Rating Scale (BFMDRS) protocol that will be rated by two movement disorder experts	10 min	Clinician scored video assessment (quantitative when combined with disability scale score)

*BFMDRS* Burke-Fahn-Marsden Dystonia Rating Scale

#### Eligibility criteria

Participants will need to be enrolled in the Global Myoclonus Dystonia Registry and Dystonia Non-Motor Symptom Study such that the presence of symptoms of anxiety and/or depression will have been established for participation in this study. More specific inclusion and exclusion criteria for this study are shown in Table 2.

**Table 2:** Inclusion and exclusion criteria

Inclusion criteria	Exclusion criteria
Individuals > 18 years of age	Individuals < 18 years of age
Diagnosis of focal cervical dystonia	Diagnosis of another dystonic disorder
Receiving ongoing neurotoxin treatment	Lacking capacity
Ability to read and write fluently in English	Treatment with deep brain stimulation
Access to the internet using either a handheld or desktop-based device	Previous treatment with CBT
	Other concurrent psychological therapy
	Inability to read and write in English
	No access to the internet

*CBT* cognitive behavioural therapy

### Details of intervention

The online CBT platform will be hosted by SilverCloud Health Ltd (www.silvercloudhealth.com). Participants will be directed to the study-specific website where they will enter a unique identifying code and be provided with a link to the ‘Space for Anxiety and Depression’ module of the Silver Cloud website. This is a fully validated online CBT programme, delivering evidence-based content and adapted to integrate and deliver to NICE standards of care [16, 17]. The programme content is modular (Table 3), with participants encouraged to complete one module a week (lasting 30–40 min), over 8 weeks. Participant accounts will be linked to researcher accounts to enable remote monitoring and provide a record of timing and frequency of login by the participant. Participants will also receive weekly reminders to encourage them to log into the programme. Also available as part of the programme will be a number of optional tools (Table 3) that participants are free to engage with to any degree they wish.

**Table 3:** Description of the online SilverCloud content including description of the core modules and additional available tools

Main module content	Title	Description of content
Module 1	Welcome to Silver Cloud	An introduction to the programme and how to make the most of the available resources.
Module 2	Getting Started	Demonstrates the basics of CBT and depression and anxiety. Introduces some of the key ideas of the programme.
Module 3	Understanding Feelings	This module takes a closer look at moods and emotions enabling the participant to explore different aspects of emotions, physical reactions and to see how they’re connected.
Module 4	Boosting Behaviour	Addresses how doing things a little differently can help to improve mood.
Module 5	Spotting Thoughts	Looks at the impact of unhelpful automatic thinking in relation to mood. Additional help to tune into thoughts and recognise any common thinking errors.
Module 6	Challenging Thoughts	This module takes the next step in helping to tackle distorted or overly negative thinking patterns that may impact mood.
Module 7	Managing Worry	Introduces the role of worry in anxiety, teaching techniques in how to cope with and manage worry more effectively.
Module 8	Bringing it all Together	Brings together all of the skills developed, and ideas gathered so far.
Additional tools		
Personal Journal	Allows participants to document entries relating to their experiences in relation to the modules.	
‘Understanding my situation’	Opportunity to reflect on current difficulties and their potential causes, as well as individual responses to feeling worried or low.	
‘Mood Monitor’	Opportunity to provide daily documentation of mood (Bad. Not ok, Ok, Good, Great) and the lifestyle choices made in response	
Goal Setting	Opportunity to set and document individual goals	

*CBT* cognitive behavioural therapy

The core modules of the online CBT programme ‘Space for Anxiety and Depression’ are described below:

1. *Module 1: Welcome to Silver Cloud*

This module provides the participant with an introduction to the programme, including what might be expect from the programme and how to make the most of the available resources.

2. *Module 2: Getting Started*

This provides the participant with information about depression and anxiety, as well as the application of CBT in the management of depression and anxiety. It also introduces the thoughts, feelings and behaviour (TFB) cycle within the context of anxiety and depression. Several activities/tools are also introduced, including the ‘Mood Monitor’ and the ‘Understanding My Situation’ tool.

3. *Module 3: Understanding Feelings*

Here, participants are helped to understand emotions and their function, along with the role of emotions in the TFB cycle. It engages participants to recognise complex emotions, and how these might impact physical body symptoms, as well as the impact of lifestyle choices on depression, anxiety and general wellbeing. Activities in this module include the ‘Life Choices Chart’.

4. *Module 4: Boosting Behaviour*

This module addresses the link between mood and behaviours and how some behaviours can boost mood. It aims to improve the participants’ knowledge of common behavioural traps in depression and provide guidance on how best to manage these, as well as to improve motivation during periods of low mood. It also focuses on recognising the importance of pleasurable activities and identifying activities to target distressing physical sensations associated with depression.

5. *Module 5: Spotting Thoughts*

Participants learn about the role of thoughts in depression and anxiety within the TFB cycle, learning to understand and recognise negative automatic thoughts.

6. *Module 6: Challenging Thoughts*

This module takes the next step in helping to tackle distorted or overly negative thinking patterns that may impact mood. Participants will learn about thoughts that are directly related to a change in emotion, or ‘hot thoughts’, how to recognise them, and how to challenge negative thoughts. Participants will also learn how to overcome specific thinking errors and to recognise situations where it is necessary to use thoughts to cope with difficult situations.

7. *Module 7: Managing Worry*

This focuses on the role of worry in maintaining anxiety. It teaches participants to recognise which worries are real or hypothetical and make use of strategies to identify and management these, such as a Worry Tree.

8. *Module 8: Bringing it all Together*

This final module gives the participant some preparation for coming to the end of the programme. It helps the participant recognise warning signs, how to plan for wellness as well as the importance of social support in staying well. It also helps the participant prepare for potential relapses and how to set goals for the future.

### Outcome assessments

As this study aims to determine feasibility of this approach to treatment, the outcome measures selected for inclusion have been selected to help inform future, larger trials. Baseline and outcome assessments will be completed during the study (Table 1). This extended range of non-motor and motor assessments (Table 1) will be completed either during home visits or when the participant attends the research clinic. Prior to statistical analysis, the videotaped clinical examinations of motor symptoms for each participant will be independently rated by two movement disorder experts blinded to the randomisation, with these scores included in the final analysis. To assess the feasibility of the internet-based CBT programme, we will also collect data on the number of logins to the internet-based CBT programme using the SilverCloud website. We will collect information on the number of logins and the number of modules completed at 8 weeks, 3 months and 6 months, along with the proportion of participants that used the additional resources included with the programme. We will also determine how many participants complete the 3- and 6-month follow-up and, at these appointments we will ask if they found the programme useful and if it was something that they would continue to go back to.

### Participant timeline

The participant timeline is shown in Table 4.

**Table 4:** Schema of participant activities

Activities all participants											
	First study visit *Baseline*	8-week online CBT intervention								Second study visit *Outcome*	Third study visit *Outcome*
		1	2	3	4	5	6	7	8		
Informed consent	X										
Allocation	X										
*Motor assessments*											
BFMDRS clinical examination	X									X	X
BFMDRS disability scale	X									X	X
*Psychiatric questionnaires*											
BDI	X									X	X
HAM-D	X									X	X
GAD-7	X									X	X
HAM-A	X									X	X
MINI	X									X	X
*Sleep questionnaires*											
SDQ	X									X	X
PSQI	X									X	X
ESS	X									X	X
*Pain questionnaires*											
CPAQ	X									X	X
PCS-EN	X									X	X
Short-form McGill	X									X	X
Pain Time Course Patterns	X									X	X
*Quality of life*											
SF-36	X									X	X
*Intervention group*											
*Online CBT programme*											
Module 1: ‘Welcome to SilverCloud’		X									
Module 2: ‘Getting Started’			X								
Module 3: ‘Understanding Feelings’				X							
Module 4: ‘Boosting Behaviour’					X						
Module 5: ‘Spotting Thoughts’						X					
Module 6: ‘Challenging Thoughts’							X				
Module 7: ‘Managing Worry’								X			
Module 8: ‘Bringing it all Together’									X		

Abbreviations: *BDI* Beck’s Depression Inventory, *BFMDRS* Burke-Fahn-Marsden Dystonia Rating Scale, *CBT* cognitive behavioural therapy, *CPAQ* Chronic Pain Acceptance Questionnaire, *ESS* Epworth Sleepiness Scale, *GAD-7* Generalised Anxiety Disorder-7, *HAM-A* Hamilton Anxiety Rating Scale, *HAM-D* Hamilton Depression Rating Scale, *MINI* Mini International Neuropsychiatric Interview, *PCS-EN* Pain Catastrophising Questionnaire, *PSQI* Pittsburgh Sleep Quality Index, *SDQ* Sleep Disorders Questionnaire, *SF-36* Short Form-36 Health Survey

### Withdrawal and loss to follow-up

Participants are free to withdraw from the study at any point without providing a reason. Participants will be asked to inform the research team either via phone, email, in writing or in person if they wish to withdraw. If the study team are unable to contact a participant who has consented to the study following their initial assessment, they will be considered lost to follow-up.

### Sample size

As this is a study aiming to determine the feasibility of using online, self-guided CBT for the management of non-motor symptoms in patients with cervical dystonia, no formal power calculation has been conducted. We aim to recruit 20 participants, with a target of randomising 10 participants into the treatment group and 10 participants into the control group.

### Recruitment

An invitation letter and flyer will be sent to all potential participants enrolled in the Dystonia Non-Motor Symptom Study who show increased levels of anxiety and/or depression. Increased levels of anxiety will be determined by a score of 11 or greater on the Health Anxiety Inventory (HAI), approximately the mean minus one standard deviation for individuals diagnosed with anxiety [18]. There are no standardised HAI scores to indicate levels of anxiety; therefore, we believe this will capture the majority individuals with moderate anxiety. Increased levels of depression will be determined by a score of 21 or greater on the Beck’s Depression Inventory (BDI), indicating moderate depression. Individuals with MINI screen score of greater or equal to 6, indicating they suffer from a number of psychiatric difficulties, will also be considered if they have answered yes to a question in relation to anxiety or depression. All potential participants must also meet the inclusion criteria outlined in Table 2.

Potential participants who wish to take part in the study will be asked to contact the study team, following which they will be sent a detailed study information sheet and invited to ask any questions they might have. Following this, each participant will be asked to arrange an appointment with the study team, where they will have another opportunity to ask any questions before completing the consent form and undergoing the baseline assessments.

### Assignment of intervention

#### Allocation mechanism and implementation

Randomisation will be completed in a 1:1 ratio using sealed, opaque envelopes containing a computer-generated random allocation code. Envelope selection will be made by a blinded assessor and participants given the envelope to open at their baseline assessment. We aim to randomise 10 participants to the treatment arm and 10 participants to the control arm. As our sample is small, it is possible that random allocation will lead to a sample selection bias, with the full population not being represented in each arm of the study. This will need to be considered when interpreting the results.

#### Blinding

Blinding the participant to the outcome of randomisation will not be possible due to the nature of this intervention. This could introduce bias as participants are aware if they are receiving the intervention or not, and therefore, this may affect their willingness to continue with the study, as well as their mood and state of mind. For example, participants randomised to the control arm may feel discouraged they are not receiving the intervention and therefore may withdraw from the study or demonstrate higher depression or anxiety scores. We will try to reduce this bias by conducting the baseline assessment prior to randomisation and offering participants in the control group the opportunity to try the intervention at the conclusion of the study. The assessors of the motor symptoms, undertaken by scoring of the videotaped clinical examinations, will remain blind to the participants’ random allocation.

#### Trial arms

Following randomisation, participants will be given verbal instructions related to their allocated trial arm by a member of the trial team. Participants randomised to the treatment arm will undergo an introduction to the online, guided, internet-based CBT programme by the trial team before being asked to complete weekly 30–40-min sessions of the programme over the following weeks. Participants will receive weekly prompts, either by text, telephone or email, during the course of the programme to remind and encourage them to log in. Aside from this, the participants will be able to choose their degree of engagement with the programme. Participants will undergo follow-up assessments, following the same structure as the baseline assessment at 3 and 6 months following their initial appointment to determine any longer-term effects of the online CBT programme. Participants randomised to the control group will undergo follow-up assessments at 3 and 6 months following their baseline assessments. All participants will continue to receive their routine injectable neurotoxin treatment for management of their motor symptoms throughout.

Participants in both trial arms will be directed to resources for urgent help and support if required throughout the study. This will include contact details for the trial team during regular working hours and the contact details of a national support helpline. Participants will be requested to attend their local A&E department if the support required is deemed urgent and severe. The participant’s general practitioner (GP) will also be informed of any symptom deterioration by the study team.

#### Data collection methods

We will collect non-motor symptom data using paper-based questionnaire packs, with subsequent entry into an online database. A videotaped standardised clinical examination will be used to collect data relating to the participants’ motor symptoms. Participants undergoing the online CBT intervention will also be asked if they thought the programme was useful and if it was something they would be likely to return to during their 3- and 6-month follow-up appointments. Data pertaining to participant’s use of the online CBT programme, including frequency and duration of logins, as well as use to the additional tools, will be collected via the SilverCloud platform.

#### Data management

Consent forms and questionnaires will be stored in a locked cabinet in a locked room, in a building that requires an access card for entry. Responses from the questionnaires will be imputed into an anonymised database on a secure server hosted by Cardiff University by a member of the trial team. Only members of the research team will have access to this database. All data will be re-identifiable so support can be given if there is evidence of distress, with participant identifiable information being stored in a secure database within the NHS network (PatientCare®).

Data gathered using the internet-based CBT platform will be stored on the Silver Cloud online server, to which the research team will have access. Participants will be given a unique study identifying code, so that no identifiable information need be entered. The trial team will be aware of which internet-based CBT account is linked to which participant in order to enable weekly login prompts. The trial team will also have access to data indicating the time and duration for each participant login.

#### Statistical methods

The baseline demographic characteristics of each trial arm will be described, including age, sex, clinical history, past and present treatments and social and family history. Descriptive statistics will be used for data collected to assess the feasibility of the CBT programme, including number of logins and modules completed and the proportion of participants that used the additional resources. We will not conduct comparative statistical analysis on this portion of the data as we are underpowered to do so. Questionnaires and rating scale scores will be compared using Student’s *t* test or Wilcoxon rank sum test and summarised using means (standard deviations) or medians (inter-quartile ranges) as appropriate for continuous variables. Categorical variables will be compared using chi-squared or Fisher’s exact test and summarised as frequencies with percentages (*n*, %). All statistical analyses will be performed using R statistical software.

### Monitoring

#### Data monitoring

A formal data monitoring committee (DMC) will not be convened for this study due to the feasibility nature of the study. The research will however be monitored independently by an administrative officer.

#### Harms

The safety of the online CBT programme will be monitored throughout the feasibility study by recording of all adverse events (AE). We do not anticipate any serious adverse events; however, safety will be monitored throughout and any AEs or SAEs will be recorded using a standard template and reported in line with standard operating procedures and research ethics committee requirements. The research team will review the data every 3 months to assess if there is an elevated rate of adverse events, including any increase in anxiety levels, in the treatment group. Also, if the level of withdrawal is 50% or greater, the research team will prematurely end the trial.

#### Auditing

This study is subject to inspection by The Jacque and Gloria Gossweiler Foundation as the funding organisation and may also be subject to inspection and audit by Cardiff University under their remit as sponsor.

#### Consent

Potential participants will be given as long as they wish to read the PIS, this being a minimum of 24 h. All potential participants will also be given the opportunity to consider the study and discuss it with the study team, friends or family. Informed consent will be undertaken using a paper-based consent form requiring the participant to initial each individual point to indicate their consent, as well as signing and dating the form. The study team member undertaking the informed consent will also be required to date and sign the consent form.

#### Confidentiality

The ‘NHS Code of Confidentiality’ will be followed to ensure confidentiality of personal patient data is maintained, as well as the principles of the ‘General Data Protection Regulation’ being followed by all study team members at all times. All identifiable patient data will only be accessible by the research team. All of these members of staff will have had training in ‘General Data Protection Regulation’ and GCP. Each participant will be given a unique study code for identification during analysis. For access to the Silver Cloud online CBT programme, participants in the treatment group will be given a unique identifying code that they can use to access the programme, meaning they do not have to enter any identifiable information. There is an optional section of the Silver Cloud online CBT programme that consists of free text boxes; therefore, it is possible that participants may enter identifiable information into this section. We will advise participants against entering personal/identifiable information into this section and ensure that all participants are aware that this section is not mandatory for study participation. All data collected will be re-identifiable by the research team in the event of the data suggesting there is a risk of harm to the participant. Confidentiality will only be broken if there is seen to be a risk of harm to the participant. In this setting, the study team will contact the participant’s primary caring clinician to inform them of the clinical scenario.

#### Dissemination policy

The research team will not provide any personalised feedback to participants; however, participants will be informed of the overall outcomes of the research study in writing in the form of a newsletter. The research team is committed to disseminating research findings and as a result is part of a strong interlinked working body of clinicians that allows for signposting of other clinicians and patients with cervical dystonia to the outcomes of the research. Manuscripts summarising the anonymised findings of this research study will be prepared for publication in peer-reviewed journals. The results will also be presented at scientific meetings, academic institutions and as part of public engagement events.

#### Protocol version and amendments

This publication is based on protocol version 2.1_28.01.2020. Any update protocols will be communicated to the study team via email as appropriate.

#### Sponsor information

Sponsorship for this study is provided by Cardiff University (SPON 1763-19).

## Discussion

To our knowledge, this is the first study to investigate the use of an internet-based CBT programme for management of non-motor symptoms in patients with cervical dystonia. Case studies have shown CBT can improve depression and anxiety (with a particular emphasis on health anxiety) in patients with cervical dystonia for up to 6 months following treatment [19], with some evidence that the benefits can be seen for up to 2 years post-treatment [20]. Further studies have also provided evidence that using a cognitive behavioural approach can relieve bodily tension in patients diagnosed with dystonia [21]. To date, there has only been one proof-of-concept study investigating the benefits of CBT in patients with dystonia (*n* = 9). This cohort underwent a course of CBT combined with mindfulness following which anxiety, depression, and wellbeing scores were improved, with this effect sustained for up to 3 months post-completion of the course together with additional improvements in dystonia severity [12]. This evidence demonstrates the potential benefit of CBT for patients with dystonia for both psychological difficulties and other symptoms associated with the disorder, as well as the importance of further research into this area.

The benefit of CBT has also been shown in other movement disorders. Tremor severity can be significantly reduced in patients with functional tremor, normalising neuro-regulation in the areas of the brain associated with emotional dysregulation [22]. There is also some evidence of a positive effect of CBT in Parkinson’s disease when used to manage the non-motor symptoms of the condition [23, 24]. Dobkin et al. reported that a course of CBT had significant improvement in depression and anxiety scores as well as having a positive impact on QoL, coping and motor decline [25]. This suggests that CBT can not only be beneficial in managing anxiety and depression, but also other non-motor and motor symptoms that occur in conditions that demonstrate comorbid psychiatric symptoms.

Collectively, evidence of the positive impact of CBT in movement disorders suggest that it is a promising area to investigate as part of a management plan for patient with cervical dystonia. However, limited resources, including a shortage of suitably qualified therapists, waiting times, as well as the stigma associated with attending regular psychotherapy sessions all limit the potential widespread implementation of such an approach. As a result, the last decade has seen the development of a number of internet-based CBT programmes in an attempt to widen access and potentially implement some of these therapies in routine clinical practice.

One of the most common applications of internet-based CBT programmes is to treat anxiety and depression, with evidence supporting their success in reducing the levels of anxiety and depression in the general population [13–15]. Some are reported to derive better results in treating one symptom group over the other [26]; however, overall, the benefits appear to be largely positive [27]. There is also evidence that online programmes are comparable in their results to face-to-face treatment, with Wagner et al. reporting that at 3 months post-treatment, depression scores for patients that received the internet-based CBT remained stable while those who had received the face-to-face intervention worsened [28]. This demonstrates the ability of internet-based CBT programmes in the management of psychiatric conditions and suggests that its utilisation in other patient populations might be beneficial.

There are a number of conditions in which online CBT has been trialed. Internet-based CBT has been found to be effective in treating patients with insomnia [29, 30]. Insomnia is a common comorbidity in asthma and is associated with increased adverse outcomes [31]. Upon further investigation, Luyster and colleagues discovered that not only did internet-based CBT seem to improve the insomnia experiences by individual patients, but on a group level there was also an improvement in the severity of the patients’ asthma [32]. Similar improvements have been observed in the treatment of irritable bowel syndrome, with those receiving internet-based CBT showing sustained improvements compared to standard treatment over a 2-year period [33]. In the treatment of functional abdominal pain disorders in children, Lalouni et al. found significant improvements in gastrointestinal symptom severity as well as the children’s overall quality of life [34]. There are, however, instances where internet-based CBT has been found not to be effective. Helmondt et al. found that there was no positive benefit of internet-based when investigating fear of cancer resurgence [35], although this was with use of a self-guided programme with no additional support. Other barriers to the success of internet-based CBT include non-engagement with the programme due to lack of time, lack of necessary IT skills, or lack of motivation to change. However, overall evidence to date suggests that the use of internet-based CBT for the treatment of chronic health problems is positive, aiding the reduction in levels of anxiety and depression as well as condition-specific outcomes [36].

Recognition of psychiatric comorbidity in dystonia is now well established, although guidelines and best practice for management of these remain to be established [3–5]. Psychiatric difficulties have been shown to have an impact on both psychological and physical QoL, which in some cases is greater than the cervical dystonia motor severity [6, 37]. Therefore, given the success of the intervention in other conditions, internet-based CBT represent a promising avenue of investigation. The improved accessibility, low cost, and reduced stigma compared to attending face-to-face therapy sessions contribute to make this an attractive management option. Success in demonstrating the feasibility of an internet-based CBT programme would have a significant impact on the clinical care delivery for patients with cervical dystonia, as well as having the potential to maximise the use of healthcare resources and widen access to effective treatment.

## Data Availability

Fully anonymised data produced as a result of this study can be made available to other researchers upon request to the Chief Investigator.

## Abbreviations

A&E: Accident and emergency
AE: Adverse events
BDI: Beck’s Depression Inventory
BFMDRS: Burke-Fahn-Marsden Dystonia Rating Scale
CBT: Cognitive behavioural therapy
CPAQ: Chronic Pain Acceptance Questionnaire
DBS: Deep brain stimulation
DMC: Data Monitoring Committee
ESS: Epworth Sleepiness Scale
GAD-7: Generalised Anxiety Disorder-7
GCP: Good clinical practice
GP: General practitioner
HAM-A: Hamilton Anxiety Rating Scale
HAM-D: Hamilton Depression Rating Scale
HAI: Health Anxiety Inventory
IT: Information technology
MINI: Mini International Neuropsychiatric Interview
MMS: Modified Mini Screen
NHS: National Health Service
NICE: National Institute for Health and Care Excellence
NMS: Non-motor symptoms
PCS-EN: Pain Catastrophising Questionnaire
PIS: Participant Information Sheet
PSQI: Pittsburgh Sleep Quality Index
QoL: Quality of life
SAE: Serious adverse events
SDQ: Sleep Disorders Questionnaire
SF-36: Short Form-36 Health Survey
TFB: Thoughts, feelings, and behaviour
